# Routine measurement in low back pain; towards a pragmatic patient-reported productivity cost outcome measurement using the institute for medical technology assessment productivity cost questionnaire

**DOI:** 10.1007/s10198-025-01756-9

**Published:** 2025-02-21

**Authors:** Adekunle Z. Ademiluyi, Antoinette D. I. van Asselt, Michiel F. Reneman

**Affiliations:** 1 University of Groningen, Groningen, The Netherlands; 2 Department of Epidemiology, University of Groningen, University Medical Center Groningen, Groningen, The Netherlands; 3 Department of Health Sciences, University of Groningen, University Medical Center Groningen, Groningen, The Netherlands; 4 Department of Rehabilitation Medicine, University of Groningen, University Medical Center Groningen, Haren-Groningen, The Netherlands

**Keywords:** Groningen spine cohort, Short-form, Productivity cost questionnaire, SEM; Standard error of measurement, R; correlation co-efficient, ICC; intra-class correlation co-efficient, JEL-I19

## Abstract

**Purpose:**

The iMTA productivity cost questionnaire (iPCQ) has been recommended as a measurement tool for productivity cost, however, its use in routine care is hindered by the length of this questionnaire (18 questions). This study developed and tested a short-form (SF-) iPCQ.

**Method:**

A secondary analysis of the Groningen Spine Cohort’s baseline data from patients with low back pain referred for tertiary care was performed. Six SFs were evaluated against the comprehensive iPCQ. Spearman correlation (r), intraclass correlation coefficient (ICC, agreement), standard error of measurement (SEM), and Bland-Altman’s plot tested the congruence of the SFs with the comprehensive iPCQ.

**Results:**

The sample consisted of 1220 patients with low back pain. The SF version with the highest correlation (SF-3; 7 items) with the comprehensive iPCQ had *r* = 0.99, ICC = 0.99, SEM = 295, while the SF with the least number of items (SF-6; 5 items) had *r* = 0.84, ICC = 0.91, SEM = 2063. The mean productivity cost estimates of SF-3 and SF-6 were €3414 (95% CI: 3036–3791) and €3333 (95% CI: 2970–3696) respectively while that for the comprehensive iPCQ amounted to €3456 (95% CI: 3189–3720).

**Conclusion:**

A SF with seven questions was developed with a high agreement with the comprehensive iPCQ. Initial clinimetric testing was satisfactory. Further assessment is recommended.

## Introduction

Low Back Pain (LBP) constitutes a significant public health problem with high accompanying societal costs, including productivity cost as the main cost driver [1–11]. Globally, LBP entails substantial direct costs (utilization of resources for clinical care) and indirect costs (associated with paid and unpaid production loss due to illness) [12, 13]. Including indirect costs in total costs for economic evaluations that take a societal perspective is pertinent if we envisage allocating scarce health resources efficiently [14, 15]. However, the existing measures of productivity cost are often lengthy and comprehensive, posing challenges in their practical application and integration into routine clinical assessments.

Productivity cost refers to the “cost associated with production loss and replacement cost due to illness, disability, and death of productive persons, both paid and unpaid” [12]. Presently, there is no gold standard measure for productivity cost [16–18]. The Institute for Medical Technology Assessment (iMTA) Productivity Cost Questionnaire (iPCQ), recommended by the Dutch guideline for health economic evaluation, was developed to assess productivity cost [15, 16, 19, 20]. The questionnaire consists of 18 items in total, exploring three domains (absenteeism, presenteeism, and unpaid work) of cost due to production loss. These domains are commensurate with a consensus that productivity costs should encompass both paid and unpaid work [15, 21].

Economic evaluation adopting a societal perspective should include productivity cost [22–28]. However, a majority of evaluations still exclude it, even those that state to take a societal perspective [22–26]. The reasons for excluding productivity costs in these evaluations are ambiguous; however, several reports suggest that the lack of international guidance and standardized measures is a possible factor [16, 18]. Furthermore, as most productivity cost questionnaires are patients’ self-completed questionnaires, the length of these measures could affect data collection and adherence. Existing tools, while valuable in their comprehensiveness, often prove impractical in real-world settings due to their length and complexity. This limitation hampers their widespread adoption in both clinical and research settings. Studies have shown that shorter patient-reported outcome measures could improve uptake and quality, and recede patient’s burden for completion [29]. Despite these findings, no brief validated productivity cost measuring instrument is presently available.

This research aims to develop a concise, user-friendly short-form measure for assessing productivity costs in patients. Striking a balance between brevity and accuracy, our goal is to create a tool seamlessly integrable into clinical practice, offering valuable insights into the economic consequences of LBP without imposing undue burdens on patients or healthcare professionals.

The significance of this study lies in its potential to enhance the efficiency of productivity cost assessment. A streamlined and accessible measure would not only facilitate broader adoption in clinical practice but also contribute to the development of targeted interventions aimed at minimizing the economic impact of illnesses on individuals and society at large.

## Methods

### Study design and participants

Cross-sectional design and baseline data were used from the Groningen Spine Cohort (GSC): a 10-year longitudinal study that commenced in 2015 at a multi-disciplinary secondary and tertiary care Spine Center in the north of the Netherlands [11]. A total of 1,502 patients aged 18 to 65 years with low back pain (LBP), with or without leg pain, were initially included in the study. However, 282 participants were excluded as they did not meet the inclusion and exclusion criteria, primarily due to incomplete data, including data vital for this study. Detailed in- and exclusion criteria are described elsewhere [11, 30]. Informed consent was obtained. A waiver was provided by the Medical Research and Ethics Committee of the University Medical Center Groningen, the Netherlands.

## Measures

### Productivity cost

Productivity cost was assessed with the Institute for Medical Technology Assessment (iMTA) Productivity Cost Questionnaire (iPCQ) [15, 20]. It contains 18 questions that evaluate absenteeism (productivity losses due to sick leave), presenteeism (reduced productivity at work from illness) and unpaid work losses with a recall period of four weeks between subsequent use. The questionnaire is sectioned into three parts;

- Six general questions.

A1. On what date are you completing this questionnaire?

A2. What is your date of birth?

A3. What is your gender?

A4. What is the highest level of education you have completed?

A5. What is your occupation?

A6. Do you have paid work?

- Three questions about paid work.

Q1. What is your occupation?

Q2. How many hours a week do you work? Add together all the hours for which you are paid.

Q3. How many days a week do you work?

- Nine questions on productivity loss.
  Absenteeism: short- and long-term absenteeism (three questions).

Q4. Have you been absent from your work in the past 4 weeks because you were ill? *This evaluates short-term absenteeism*.

Q5. Have you been absent from your work because of being ill for longer than the entire period of 4 weeks? *This depicts long-term absenteeism*.

Q6. When did you report being ill?

Presenteeism (three questions).

Q7. Have there been days over the past 4 weeks when you worked but suffered from physical or psychological problems during your work?

Q8. On how many working days have you suffered from physical or psychological problems during your work? Just count the working days over the past 4 weeks.

Q9. On the days when you were suffering from problems, perhaps you were not able to do as much work as normal. On those days, how much work could you do on average? Look at the numbers below. A 10 means that you were able to do just as much as normal on those days. A 0 means that you were not able to do anything on those days.

Unpaid work (three questions).

Q10. Have there been any days on which you were able to do less unpaid work because of your physical or psychological problems? This relates to days over the past 4 weeks.

Q11. On how many days was this the case? Only count the days in the past 4 weeks.

Q12. Suppose that someone, for example, your partner, a family member, or an acquaintance, has helped you on these days. And did all that unpaid work for you, that you could not do. How many hours, on average, was that person busy with it on these days?

Sufficient feasibility, reliability, content validity and construct validity were established in patients with musculoskeletal disorders [21, 31–33]. This questionnaire can be requested through the iMTA at Erasmus University Rotterdam.

### National institutes of health minimal dataset

The National Institutes of Health (NIH) minimal dataset includes items on patient characteristics, medical history, and self-reported symptoms and functioning [34]. This depicted the demographic data.

### Data pre-processing

#### Calculation of productivity cost using iPCQ

##### Absenteeism

The cost of absenteeism is calculated by multiplying the working hours missed in the last four weeks by the cost of production per hour according to the Dutch guideline for economic evaluation (2015; €34.96 for average, €31.80 for women, and €38.10 for men) [19]. If the time the patient has been absent from work exceeds 4 weeks (long-term absence), then an estimate of the number of workdays missed (in calendar days) was calculated by subtracting the date the patient reported in sick from the date the iPCQ was filled. The friction cost method (which assumes that another worker will fill in after 85 days) was applied to estimate the cost associated with absenteeism [35]. The iPCQ does not differentiate between part-time and full-time sick leave.

##### Presenteeism

The cost of loss in productivity due to inefficiency of being sick at work was assessed by multiplying the number of workdays impaired (4 weeks recall period), the number of working hours per day, efficiency ratio [1*(efficiency score selected on a scale of 0–10 by the respondent/10)], and the cost of production loss according to the Dutch guideline for economic evaluation in health care research (2015; €34.96 for average, €31.80 for women, and €38.10 for men) [19].

##### Unpaid work

The productivity loss associated with the inability to do unpaid work was estimated by multiplying the number of days missed by the number of hours of help needed per day to make up for it. Multiplying an hourly rate (according to the Dutch guideline for economic evaluation, €14.08) for those chores by the hours of productivity loss accrued, depicted the cost [19].

### Statistical analysis

#### Missing data handling

Missing data on working hours and working days were managed with multiple imputations using SPSS. A minimum and a maximum number of working days (between 1 and 5 days/week) and working hours (between 2 and 40 h) were set based on the possible minimum and maximum inputs attainable. Age, sex, level of education, and average work-hours per week were only predictors while the number of working days per week and number of working hours per week were both imputed and predictors in the model. Five imputed datasets were created.

### Shortening of iPCQ (Ad-hoc analysis)

#### Attempt

**Fig. 1:**
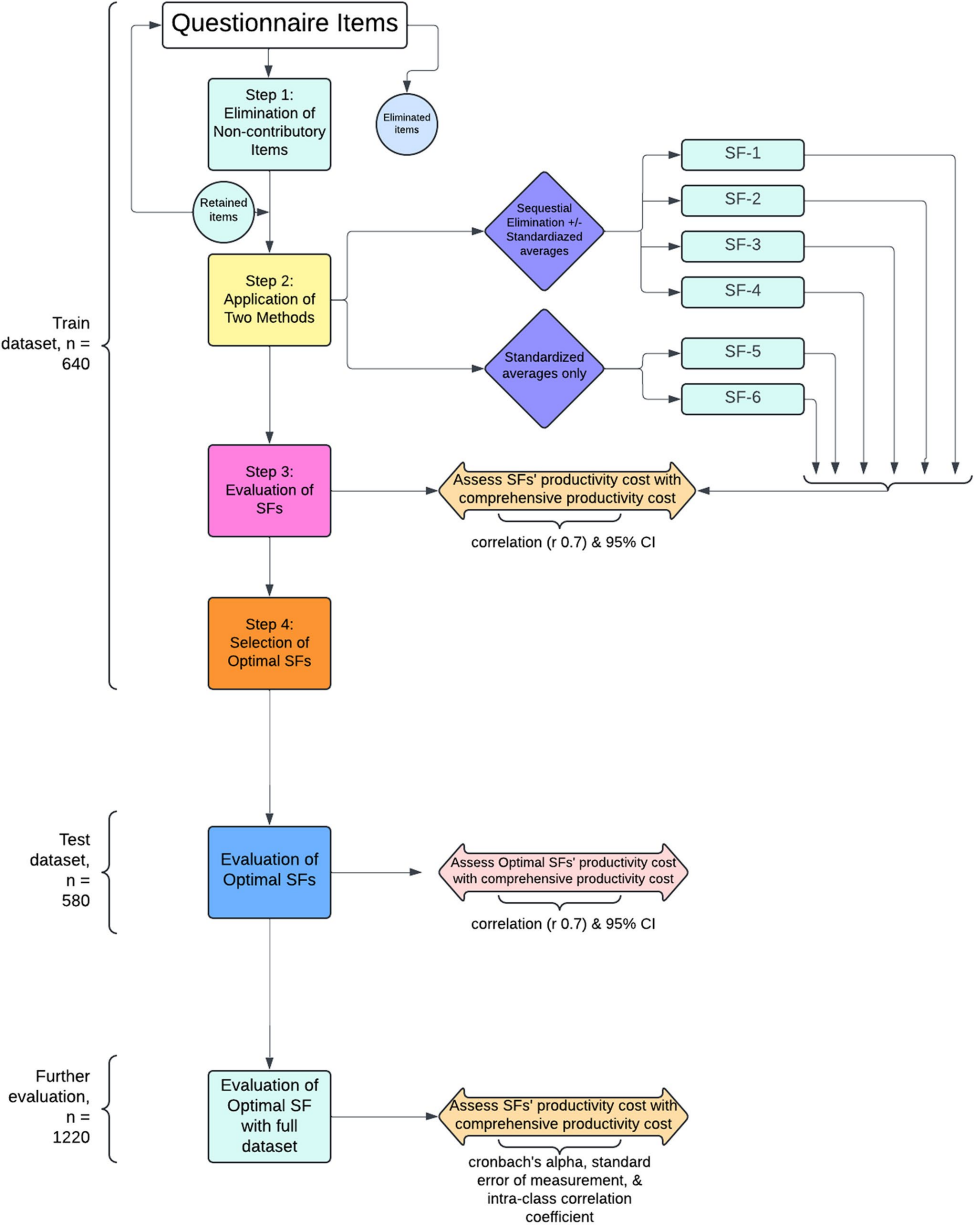
Schematic diagram of the shortening of iPCQ

The iPCQ consists of three parts, and a progressive shortening process was applied. Firstly, the entire baseline data set was randomly split into two subsets: the train dataset and the test dataset. Splitting of the dataset was done to train the short-form questionnaire on a subset (train data) and then evaluate its performance on another subset (test data). This evaluates how well the short-form questionnaire generalized to new data. The differences in participant characteristics between the datasets were small and non-significant. As such, we assumed the results should be similar between train and test data. This is shown in Fig. 1.

#### Train data

The following steps were performed;

##### Step 1

The initial phase involved refining the questionnaire by eliminating items that did not contribute to calculating productivity costs. This process targeted redundant questions - having been covered by previous questionnaires or earlier data collection efforts (items A1 to A5) - or provided limited value as standalone items, particularly dichotomous questions (items Q7). The details of these eliminations are presented in Table 1 as ‘x’.

**Table 1:** Description of the items in each short-form iPCQ

Comprehensive iPCQ	SF-1	SF-2	SF-3	SF-4	SF-5	SF-6
**General Questions**						
A1	x	x	x	x	x	x
A2	x	x	x	x	x	x
A3	x	x	x	x	x	x
A4	x	x	x	x	x	x
A5	x	x	x	x	x	x
A6	Remained *	Remained *	Remained *	Remained *	Remained *	Remained *
**Questions about Paid work**						
Q1	x	x	x	x	x	x
Q2	Remained *	Replaced with a Dutch-population based average	Remained *	Replaced with a Dutch-population based average	Replaced with a Dutch-population based average	Replaced with a Dutch-population based average
Q3	x	x	x	x	x	x
**Productivity loss modules**						
-Absenteeism						
Q4	x	x	Remained *	Remained *	Remained *	x
Q5	x	x	x	x	x	x
Q6	Remained *	Remained *	Remained *	Remained *	Remained *	Remained *
-Presenteeism						
Q7	Merged (Q7 + Q8) *	Merged (Q7 + Q8) *	Merged (Q7 + Q8) *	Merged (Q7 + Q8) *	Merged (Q7 + Q8) *	Merged (Q7 + Q8) *
Q8						
Q9	Remained *	Remained *	Remained *	Remained *	Remained *	Remained *
-Unpaid work						
Q10	Merged (Q10 + Q11) *	Merged (Q10 + Q11) *	Merged (Q10 + Q11) *	Merged (Q10 + Q11) *	Replaced with a Dutch-population based average	Replaced with a Dutch-population based average
Q11						
Q12	Remained *	Replaced with a Dutch-population based average	Remained *	Replaced with a Dutch-population based average	Replaced with a Dutch-population based average	Replaced with a Dutch-population based average
Total items^$^ = 18	7	6	7	6	5	4

iPCQ, institute of medical technology and assessment productivity cost questionnaire; SF, short-form; x, excluded from the total number of items; * included as item in total number; ^$^ total number of items includes all items marked by *; Population-based averages, Dutch-population based averages are generally available from reputable sources

##### Step 2

Next, the following approach was employed to further streamline the questionnaire.

The approach involved the systematic removal of items related to the three domains of productivity: absenteeism, presenteeism, and unpaid work. The objective was to create a concise tool for assessing productivity costs while reducing the number of questionnaire items. Reduction was achieved by, for certain items, using Dutch population-based standardized averages, or excluding them entirely. This process resulted in the development of six short forms (SFs 1–6). The SFs varied in which items were included, and in what way. SFs 1 and 2 reconstructed paid work absenteeism by calculating the difference in days between the date the questionnaire was completed and the date the respondent reported sick. In addition, SF2 applied population-based averages for the number of hours paid work per week, and the number of hours unpaid work lost per day. SFs 3 and 4 were similar to SF 1 and 2, respectively, but included Q4 on absence of paid work. In SF5 and SF6, also the number of days of unpaid work lost was replaced by a population average.

The specifics of all six configurations are detailed in Table 1.

##### Step 3

Subsequently, the average productivity costs and the correlations of each SF were evaluated in comparison to the standard method. This evaluation aimed to determine the alignment of SF-derived values with those obtained using the standard method, focusing on proximity within the 95% confidence interval (CI) and a correlation coefficient threshold of *r* ≥ 0.7. The assessment identified shortening methods that maintained a high correlation with the standard method as the most effective.

##### Step 4

Short Forms that demonstrated strong alignment with the standard method—based on the correlation, 95% CI of average productivity costs, and shortest number of items—were selected for further validation using the test dataset.

#### Test data

The shortening methods obtained in step 4 above were used to assess the productivity cost in this data set. The productivity cost from the SFs was correlated with the result of the iPCQ using Pearson or Spearman correlation test (p-value ≤ 0.05 and $\:r$≥0.7) and 95% CI of the mean productivity cost. The shortening method with the closest proximity to the iPCQ in terms of the mean, correlation coefficient and shortest number of items included was then chosen as the new SF.

### Further evaluations

Using the SFs selected in the test data, productivity cost at baseline of all the observations in the full dataset was computed and evaluated against the comprehensive iPCQ at baseline. Cronbach’s alpha, intra-class correlation coefficient (consistency and agreement $\:r\ge\:0.7$ is considered satisfactory), and standard error of measurement (SEM = √Mean square_error,_ obtained from ANOVA) of the SFs were evaluated against the comprehensive iPCQ. Data were analyzed with SPSS statistical package version 28.

## Results

Characteristics of 1220 participants from the Groningen Spine Cohort are presented in Table 2. Differences in patient characteristics between the train and test data were small and non-significant.

**Table 2:** Participants characteristics

		Total (*n* = 1220)	TRAIN DATA (*n* = 640)	TEST DATA (*n* = 580)
Age, mean (SD)		46.4 (12.53)	46.4 (12.45)	46.56 (12.61)
Sex, n (%)	Male	528 (43.3)	282 (44.1)	246 (42.4)
Education, n (%)	No Education	17 (1.7)	8 (1.3)	9 (1.9)
	Low Education	422 (34.6)	217 (33.9)	205 (35.3)
	Middle Education	400 (32.8)	212 (33.2)	188 (32.4)
	High Education	283 (23.2)	160 (25)	123 (21.2)
	Others	98 (8)	43 (6.7)	55 (9.5)
Employment, n (%)	Not working	386 (31.6)	197 (30.8)	189 (32.6)
	Working	834 (68.4)	443 (69.2)	391 (67.4)
	No Sick leave	358 (29.3)	190 (29.7.7)	168 (29)
	Partially Sick leave	246 (20.2)	129 (20.2)	117 (20.2)
	Full-time Sick leave	230 (18.9)	124 (19.4)	106 (18.3)
Work	Working hours/week, Mean ± SD	31.1 (12.4)	31.3 (12.4)	30.8 (12.5)
	Working days/week, Mean ± SD	3.9 (1.6)	3.9 (1.6)	4 (1.6)
	Workability, (0–10) median (IOR)	4 (5)	4 (5)	4 (5)

IQR, interquartile range; N, number of patients

### Shortening

#### Train data

Using the comprehensive iPCQ in the Train dataset, the participants accrued a mean total productivity cost of €3491 (95% CI: 3121–3860). The mean total productivity costs of SFs; 1, 3, and 5 were €3214, €3420, and €3777, respectively. The methods including shortened unpaid work (SFs; 2, 4, and 6) resulted in a mean total productivity cost of €2759, €2964, and €3322 respectively. Costs due to absenteeism, presenteeism and unpaid work are presented in Table 3. SF-2 and SF-3 in comparison to other short forms, showed the highest association with the standard method of computing productivity cost with a correlation coefficient of 0.99 and 0.90 respectively. SF-6 showed the shortest number of items with the highest correlation compared to other short forms. SFs; 2,3, and 6 were then explored in the Test dataset.

**Table 3:** Comprehensive productivity cost versus short-forms

	**TRAIN DATASET**						
	**Standard method**	**SF-1**	**SF-2**	**SF-3**	**SF-4**	**SF-5**	**SF-6**
	**Mean Cost** **(95% CI) €**	**Mean Cost** **(95% CI) €**	**Mean Cost** **(95% CI) €**	**Mean Cost** **(95% CI) €**	**Mean Cost** **(95% CI) €**	**Mean Cost** **(95% CI) €**	**Mean Cost** **(95% CI) €**
Absenteeism (*N* = 213)	5995 (5233–6757)	5701 (4915–6487)	5701 (4915–6487)	5701 (4915–6487)	5701 (4915–6487)	6742 (6046–7437)	6742 (6046–7437)
Presenteeism (*N* = 338)	921 (818–1024)	583 (513–652)	583 (513–652)	971 (855–1087)	971 (855–1087)	993 (891–1095)	993 (891–1095)
Unpaid Work (*N* = 428)	1509 (1306–1712)	1509 (1306–1712)	828 (787–870)	1509 (1306–1712)	828 (787–870)	1509 (1306–1712)	828 (787–870)
Mean Total Productivity cost (*N* = 640)	3491 (3121–3860)	3214 (2849–3580)	2759 (2419–3099)	3420 (3051–3788)	2964 (2619–3309)	3777 (3405–4150)	3322 (2970–3674)
Standard Method r (p value) (*N* = 640)	1	0.99 (< 0.0005)	0.90 (< 0.0005)	0.99 (< 0.0005)	0.90 (< 0.0005)	0.98 (< 0.0005)	0.88 (< 0.0005)
	**TEST DATASET**						
	**Standard method**		**SF-2**		**SF-3**		**SF-6**
	**Mean Cost** **(CI) €**		**Mean Cost** **(95% CI) €**		**Mean Cost** **(95% CI) €**		**Mean Cost** **(95% CI) €**
Absenteeism (*N* = 175)	6412 (5614–7210)		6191 (5367–7015)		6191 (5367–7015)		7252 (6507–7996)
Presenteeism (*N* = 316)	1063 (945–1181)		702 (614–791)		1170 (1023–1318)		1148 (1024–1273)
Unpaid Work (*N* = 394)	1326 (1141–1511)		783 (738–828)		1326 (1141–1511)		783 (738–828)
Mean Total Productivity cost, (IOR) (*N* = 580)	3415 (3032–3797)		2783 (2430–3136)		3407 (3020–3794)		3346 (2970–3721)
Standard Method r (p value) (*N* = 580)	1		0.92 (< 0.005)		0.99 (< 0.00005)		0.90 (< 0.005)

#### Test data

In Table 3 below, 580 participants in the dataset accounted for the total productivity cost with a mean of €3415 (95% CI: 3032–3797) using the comprehensive iPCQ. Other parameters can be seen in Table 3.

### Further evaluation

The mean total productivity cost of all the sample participants using the comprehensive iPCQ amounted to €3456 (95% CI: 3189–3720) respectively. Furthermore, the Cronbach’s alpha and ICC agreement and ICC consistency for SFs are shown in Table 4. In Table 1, the number of items and domains of the newly shortened productivity cost questionnaires were presented compared to the comprehensive iPCQ.

**Table 4:** Evaluation of the short-form and comprehensive on all the participants (*n* = 1220)

Item	Inter-item ICC (*p*)	Cronbach’s alpha (*p*)	ICC_agreement_ (95% CI)	ICC_consistency_ (95% CI)	SEM
SF-2	0.94 (< 0.005)	0.97 (< 0.005)	0.96 (0.94–0.97)	0.97 (0.963–0.972)	1122
SF-3	0.99 (< 0.005)	0.99 (< 0.05)	0.99 (0.996–0.998)	0.99 (0.995–0.998)	295
SF-6	0.84 (< 0.005)	0.91 (< 0.0005)	0.91 (0.90–0.92)	0.91 (0.90–0.92)	2063

ICC, Intra-class correlation co-efficient; SEM, Standard error of Measurement

In this study, multiple imputation was performed to handle missing data across five imputed datasets. It is important to consider that the assumptions underlying the imputation process can influence the results. To assess the robustness of our findings, we examined the variability across the five imputed datasets. No significant discrepancies were observed between the results generated from the individual datasets. However, sensitivity analyses were not conducted to further explore how different imputation models (e.g., varying assumptions about the distribution of missing data) might affect the outcomes. Future research could incorporate such analyses to provide a more detailed understanding of the influence of imputation assumptions. The consistency observed across the five imputed datasets supports the reliability of the presented results, although we acknowledge that exploring alternative imputation strategies might provide additional insights.

## Discussion

Of the six methods explored, SF-3, although not the shortest, had the highest ICC (0.998) with the comprehensive iPCQ and the smallest standard error of measurement. Mean square_error_ was used instead of intraclass correlation coefficient in the estimation of the standard error of measurement because it is not influenced by between-subject variations but focuses on within-subject variations which are pertinent to our study. Furthermore, these findings would be difficult to compare with other studies because these studies explored the components (absenteeism, presenteeism, and unpaid work) of productivity cost in their assessment rather than the total productivity cost of each participant [15, 20, 32].

Analyzing the ICCs of this study with other studies is difficult because other studies used time as a factor of the reliability (test-retest reliability) [20, 32]. Also, studies on productivity cost assessment, especially studies using iPCQ, are scarce, despite the relevance of societal cost assessment in economic evaluation. This could be due to the use of sick leave registries and the absence of standardized measures [16, 18, 36]. However, not all countries have these types of registries and policymakers are moving towards the use of validated measures to assess productivity costs [15, 16, 19].

Compared to the other short forms, SF-3 contained the most items similar to those in the comprehensive questionnaire, which likely explains the strong correlation observed between them. Despite the replacement of some items in the other short forms, significant correlations with the comprehensive questionnaire were maintained. However, an increase in the standard error of measurement (SEM) was noted with additional replacements. For example, SF-6, which had the fewest items due to more items being replaced by population-standardized averages, exhibited higher SEM than SF-2. The mean productivity cost derived from SF-3 and SF-6 was close to that of the comprehensive questionnaire. This indicates that further replacements may be possible in SF-3, although such changes might reduce measurement accuracy. This study represents an initial step towards further validation and the exploration of additional measurement properties of SF-3, in comparison with the other short forms, which also showed significant correlations and fewer items. Across all the short forms examined, some items were identified as repetitive of information previously obtained from respondents and were thus excluded. In cases where such information is unavailable, these items can be included if applicable.

### Strengths and limitations

A notable strength of our study lies in its pioneering initiative to shorten the iMTA Productivity Cost Questionnaire (iPCQ). The absence of prior attempts in the literature highlights the innovative nature of our work. Through the introduction of short-form measures like SF-3, we contribute a novel tool for efficient productivity cost assessment. However, this lack of precedent also serves as a limitation, impeding direct comparisons and contextualization of the effectiveness and impact of our short-form measures. Addressing this limitation in future research will enhance the broader understanding and validity of our innovative approach. A significant strength of this study is its large sample size, which enhances the reliability and generalizability of our findings on the feasibility of a shorter questionnaire to assess productivity costs. Moreover, the study population includes patients with LBP receiving secondary or tertiary care, many of whom are employed and face productivity-related challenges due to their condition. This makes the dataset particularly rich and relevant for evaluating a streamlined approach to productivity cost assessment in a working-age population. In addition, the objective of the study was to reduce the number of items from the comprehensive iPCQ, without introducing any novel items. All items utilized in this exploration were derived from the existing comprehensive iPCQ. This represents a significant advancement in developing a potentially shorter and more efficient form of the productivity cost questionnaire. A potential limitation in the study arises from the testing of a generic questionnaire within a highly specific sample comprising individuals with low back pain referred to tertiary care. This narrowed sample may impact the generalizability of the findings to broader populations. In addition, one limitation of this study is that sensitivity analyses were not performed to investigate the impact of different imputation models, such as varying assumptions about the distribution of missing data, on the outcomes. Incorporating such analyses in future research could enhance understanding of how imputation assumptions influence the results. While the consistency observed across the five imputed datasets supports the reliability of the findings, the potential benefits of exploring alternative imputation strategies remain unexamined and could offer further valuable insights. Furthermore, the reliability assessed was a factor of the items removed or replaced and not the time interval as other clinimetric studies have done [32]. Also, these short forms in this study were not directly tested by the patients. Though the short forms appear easier to complete, we must test whether they would be suitable for routine outcome measurement. In addition, a limitation of the study is that it exclusively assessed the total score of productivity cost and did not investigate the three individual components. This focus on the overall productivity cost score may restrict the depth of insights into specific aspects, potentially overlooking nuances present within the separate components of productivity cost. Mitigating this constraint in future studies will contribute to a more comprehensive understanding and validation of our novel approach.

## Conclusion

A shortened productivity cost questionnaire has been created with a good correlation, reliability, and agreement with the comprehensive iMTA productivity cost questionnaire. The current study provides a foundation for future research into developing pragmatic SF measures for productivity cost.

## Appendix

The details about the full content of the questionnaire can be requested from the website: https://www.imta.nl/.
